# Addressing transcranial electrical stimulation variability through prospective individualized dosing of electric field strength in 300 participants across two samples: the 2-SPED approach

**DOI:** 10.1088/1741-2552/ac9a78

**Published:** 2022-10-28

**Authors:** Sybren Van Hoornweder, Kevin A Caulfield, Michael Nitsche, Axel Thielscher, Raf L J Meesen

**Affiliations:** 1 REVAL—Rehabilitation Research Center, Faculty of Rehabilitation Sciences, University of Hasselt, Diepenbeek, Belgium; 2 Brain Stimulation Laboratory, Department of Psychiatry, Medical University of South Carolina, Charleston, SC, United States of America; 3 Department of Psychology and Neurosciences, Leibniz Research Centre for Working Environment and Human Factors, Dortmund, Germany; 4 Department of Neurology, University Medical Hospital Bergmannsheil, Bürkle de la Camp-Platz, Bochum, Germany; 5 Section for Magnetic Resonance, Department of Health Technology, Technical University of Denmark, Kgs Lyngby, Denmark; 6 Danish Research Centre for Magnetic Resonance, Centre for Functional and Diagnostic Imaging and Research, Copenhagen University Hospital Amager and Hvidovre, Copenhagen, Denmark; 7 Movement Control and Neuroplasticity Research Group, Department of Movement Sciences, Group Biomedical Sciences KU Leuven, Leuven, Belgium

**Keywords:** electric field (E-field) modeling, transcranial electrical stimulation (tES), transcranial direct current stimulation (tDCS), finite element method (FEM), noninvasive brain stimulation, computational dosimetry

## Abstract

*Objective*. Transcranial electrical stimulation (tES) is a promising method for modulating brain activity and excitability with variable results to date. To minimize electric (E-)field strength variability, we introduce the 2-sample prospective E-field dosing (2-SPED) approach, which uses E-field strengths induced by tES in a first population to individualize stimulation intensity in a second population. *Approach*. We performed E-field modeling of three common tES montages in 300 healthy younger adults. First, permutation analyses identified the sample size required to obtain a stable group average E-field in the primary motor cortex (M1), with stability being defined as the number of participants where all group-average E-field strengths ± standard deviation did not leave the population’s 5–95 percentile range. Second, this stable group average was used to individualize tES intensity in a second independent population (n = 100). The impact of individualized versus fixed intensity tES on E-field strength variability was analyzed. *Main results*. In the first population, stable group average E-field strengths (V/m) in M1 were achieved at 74–85 participants, depending on the tES montage. Individualizing the stimulation intensity (mA) in the second population resulted in uniform M1 E-field strength (all p < 0.001) and significantly diminished peak cortical E-field strength variability (all p < 0.01), across all montages. *Significance*. 2-SPED is a feasible way to prospectively induce more uniform E-field strengths in a region of interest. Future studies might apply 2-SPED to investigate whether decreased E-field strength variability also results in decreased physiological and behavioral variability in response to tES.

## 1. Introduction

Transcranial electrical stimulation (tES) is a form of noninvasive brain stimulation that propagates low intensity electrical currents through the brain, via electrodes placed on the scalp [1]. Although the electric (E-)fields generated by tES are generally too low to elicit neuronal firing, they can modulate neuronal excitability and/or entrain neuronal firing [1–4]. As a result of its ease of use, cost-effectiveness and portability, tES has become increasingly popular as both a fundamental tool to investigate the neurophysiological foundation of psychological processes, and a potential clinical therapy that promises to alter cognitive and motor behavior [5]. Although a large body of evidence has previously demonstrated the potential of tES, widely variable results present a major hurdle for routine implementation, as they give rise to small effect sizes and ambiguous conclusions [6, 7].

Per standard, tES protocols apply a fixed current intensity to each person, irrespective of individual head anatomy. However, intracranially validated modeling studies have revealed that anatomical idiosyncrasies give rise to E-field strength variations of up to 100% across persons. As the E-field generated in the brain is a cardinal physical agent of tES [8–17], next to other factors such as current direction [18], this shortcoming could account for a large part of the widely variable effects observable in tES.

**Figure 1.**
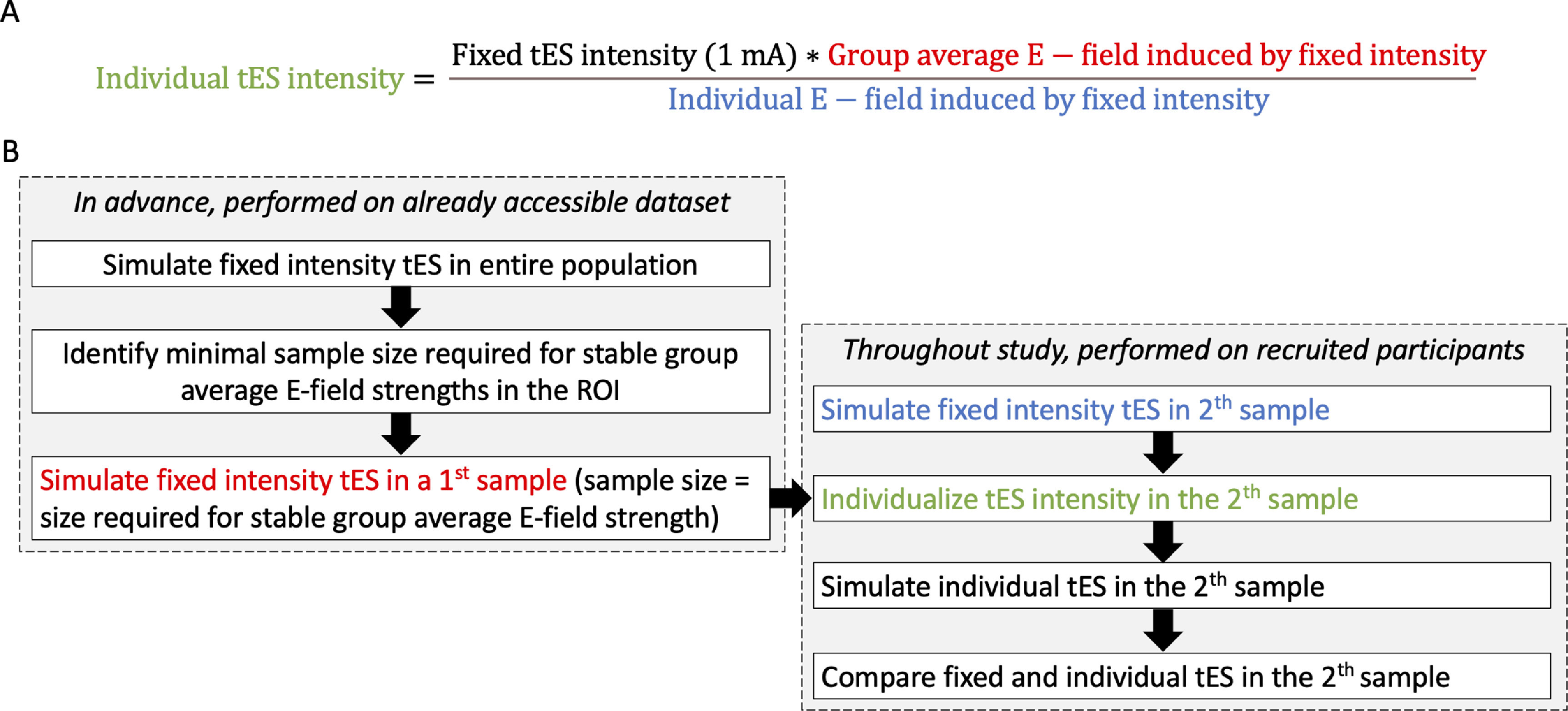
2-sample prospective electric field dosing (2-SPED). (A) Reverse-calculation formula. (B) Overview of 2-SPED approach. Through a database, all the left steps can be performed in advance. E-field = Electric field, ROI = Region of interest. tES = Transcranial electrical stimulation.

In an effort to reduce E-field strength variability across persons, a reverse-calculation method based on computational modeling dosimetry has been proposed (figure 1(A)) [13, 19–21]. This method uses the simulated E-field strength induced by fixed intensity tES in one person and on average in a group to calculate an individual stimulation intensity per person. Applying tES with this individual intensity results in uniform E-field strengths across all persons.

Although theoretically appealing, the reverse-calculation method has not yet been implemented *in-vivo*. Likely, this is due to the limiting step of determining an (average) E-field intensity to base the reverse-calculation dosing on. To date, there is not yet a consensus reached on whether there is an optimal E-field strength for dosing. Some researchers have aimed to answer this question by retrospectively determining an optimal E-field strength, but conflicting results impede unambiguous interpretation [12, 13, 15, 22]. A parallel method we propose here is to use the group average E-field induced by fixed intensity tES in a large first sample (S1) as a guide for reverse-calculation intensity dosing in a second sample (S2) (figure 1(B)). As such, we established a 2-Sample Prospective E-field Dosing (2-SPED) method. Moreover, to the best of our knowledge, the reverse-calculation method has not yet been investigated in several tES montages such as high-definition 4 × 1 tES and center-surround ring tES, despite the fact that these montages have been used in a vast array of protocols due to the presumed higher spatial focality of the E-field produced by these montages [23–25].

In summary, we aim to conceptualize and validate the 2-SPED approach in three tES montages. By leveraging the extensive Human Connectome Project MRI dataset, we can include a large number of participants to capture a wide range of anatomical idiosyncrasies. We hypothesize that the 2-SPED approach will significantly reduce peak and average ROI E-field strength variability [13].

## 2. Methods

### 2.1. Participants

In total, 300 healthy participants (150 men and 150 women) were included [26]. Inclusion criteria were persons aged 22–35 years old, Mini Mental Status Exam score ⩾29, and no history of psychiatric disorder, substance abuse, neurological and/or cardiovascular disease. Exclusion criteria were ⩾2 seizures in one’s lifetime, genetic disorders, migraine medication use in the past year, head injuries, premature birth, pregnancy, unsafe (metal) device in the body, and/or chemotherapy. The study was approved by the local ethical committee of Hasselt (approval number: CME2022/011) and was in line with the Declaration of Helsinki and its amendments.

### 2.2. Computational modeling

**Figure 2.**
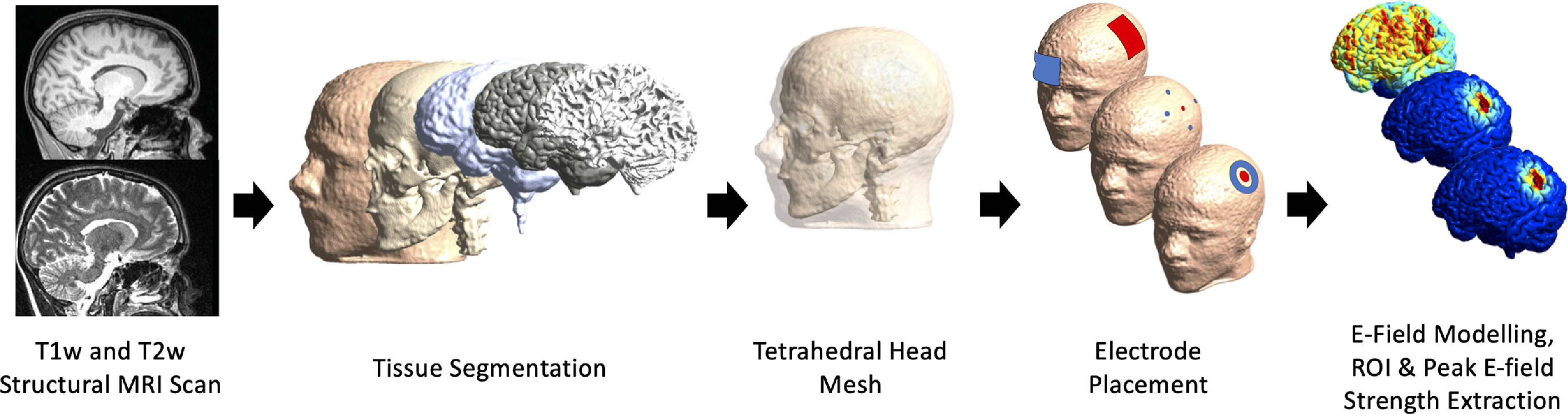
Electric field (E-field) modeling pipeline. T1-weighted and T2-weighted magnetic resonance imaging (MRI) scans from 300 participants were segmented into five tissues and an anatomically accurate tetrahedral head mesh was created. The E-fields induced by three commonly used transcranial electrical stimulation (tES) montages were subsequently simulated (from upper-left to lower-right: conventional primary motor cortex—contralateral supraorbital, 4 × 1- and center-surround tES).

In line with our previous work [27, 28], anatomical T1-weighted and T2-weighted MRI-scans were acquired with the Siemens MAGNETOM 3 T scanner (32-channel head coil). T1-weighted scans were acquired with the following parameters: TR = 2400 ms, TE = 2.14 ms, flip angle = 8°, field of view = 224 × 224 × 180 mm, voxel size = 0.7 mm^3^. T2-weighted scans were acquired with the following parameters: TR = 3200 ms, TE = 565 ms, field of view = 224 × 224 × 180 mm, voxel size = 0.7 mm^3^. MRI-scans were used to construct tetrahedral head meshes for use in E-field calculations based on the finite element method. Head model reconstruction was performed via the headreco command [29], which uses SPM12 [30] and CAT12 [31]. All head models were visually inspected to ensure accurate segmentation of the skin, bone, cerebrospinal fluid, grey matter, white matter, and eyes (figure 2). As a result of this inspection, 11 participants (6 males, 5 females) were excluded due to intersecting tissue layers, resulting in a final sample size of 289 participants.

### 2.3. Transcranial electrical stimulation

Three commonly used tES montages (figure 2) were simulated in SimNIBS (v3.2.3), which has been validated against intracranial recordings and other modeling software packages [32–34]. Conventional primary motor cortex (M1)—contralateral supraorbital (SO) tES consisted of two rectangular electrodes (4 × 5 cm) placed over C3 and FP2 (supraorbital area). The 4 × 1 montage consisted of a circular anode over C3 and four circular cathodes over FC3, C1, CP3 and C5 (0.25 cm electrode radius). Center-surround tES consisted of a circular anode (1 cm electrode radius) and a ring cathode over C3 (2 cm inner radius, 3 cm outer radius).

All montages were simulated in SimNIBS at an intensity of 1 mA. For the 4 × 1 montage, this meant that the intensity of each cathode was 0.25 mA. The following conductivities were assigned to each tissue: *σ*_white matter_ = 0.126 S/m, *σ*_grey matter_ = 0.275 S/m, *σ*_cerebrospinal fluid_ = 1.654 S/m, *σ*_bone_ = 0.01 S/m, *σ*_skin_ = 0.465 S/m, *σ*_eyes_ = 0.5 S/m, *σ*_electrode rubber_ = 29.4 S/m, *σ*_electrode gel_ = 1 S/m [35–37]. Next, E-fields in the ROI were extracted per montage and participant. The ROI was defined as a 10 mm radius sphere with the peak MNI coordinate of M1 (*x* = −37, *y* = −21, *z* = 58) transformed to subject space serving as the center point [38]. Coordinate transformation from MNI to subject space was done using the mni2subject_coord command [34].

### 2.4. Stability of a group average E-field strength in subsample 1

The current approach was based on the work of Schönbrodt and Perugini [39]. The number of participants required to achieve a stable group average E-field measure for each tES montage was calculated using bootstrapped statistics in MATLAB R2021a (The Mathworks, Inc., Natick, Massachusetts, United States). Subsamples with increasing size from 5 to 289 were randomly selected from the entire sample. The group average E-field of each subsample was calculated. This procedure was repeated 10,000 times per subsample size. Next, the 5th and 95th percentile of the entire sample was calculated, this range was defined as the corridor of stability. The subsample size at which all the group average E-field strengths ± the respective standard deviation of the subsample fell within the corridor of stability and never left it at increasing subsample sizes was defined as the point of stability. The most conservative point of stability across the 3 tES montages was used as sample size for S1. In S1, we then extracted the group average E-field strength in the ROI induced by each tES montage.

### 2.5. Testing transcranial electrical stimulation individualization in an independent subsample 2

Subsequently, 100 participants (non-overlapping with S1) were assigned to S2. Per tES montage, the group average E-field strength in the ROI of S1 was multiplied with 1 mA (i.e. the used tES intensity) (figure 1(A)). For each S2-participant and tES montage, this value was divided by the individual E-field strength induced in the ROI by the respective tES montage at an intensity of 1 mA. This resulted in an individual stimulation intensity per S2-participant and tES montage. All simulations were reconducted using the individual stimulation intensity.

In total, 600 E-field models were calculated (3 tES montages * 2 stimulation intensities * 100 S2-participants). Per model, the average E-field strength induced in M1 and robust peak E-field strength, defined as the 99th percentile of the total induced cortical E-field strength, were extracted for analyses. Inclusion of peak E-field strength gives additional information regarding the validity of 2-SPED, given that this value is not directly influenced by the reverse-calculation approach, in contrast to the average E-field strength in the ROI.

### 2.6. Statistical analyses

R (R Foundation for Statistical Computing, Vienna, Austria) and RStudio (RStudio Team, Boston, Massachusetts, United States) were used for the statistical analyses [40, 41]. Descriptive statistics (average, standard deviation, variation coefficient [VC], minimum, maximum and range) were calculated to examine E-field strength induced by fixed and individual intensity tES. Moreover, differences in ROI and peak E-field variability between fixed versus individual tES were inspected through inferential statistics. To this end, the modified Pitman–Morgan test, a pairwise test based on Spearman’s rank correlations instead of Pearson’s R correlations, was used [42]. This modified test was used due to its superior robustness against Type 1 errors in case of deviation from normality. The significance level was set to alpha = 0.05.

## 3. Results

Results are noted as average ± standard deviation unless stated otherwise.

### 3.1. Stability analyses

**Figure 3.**
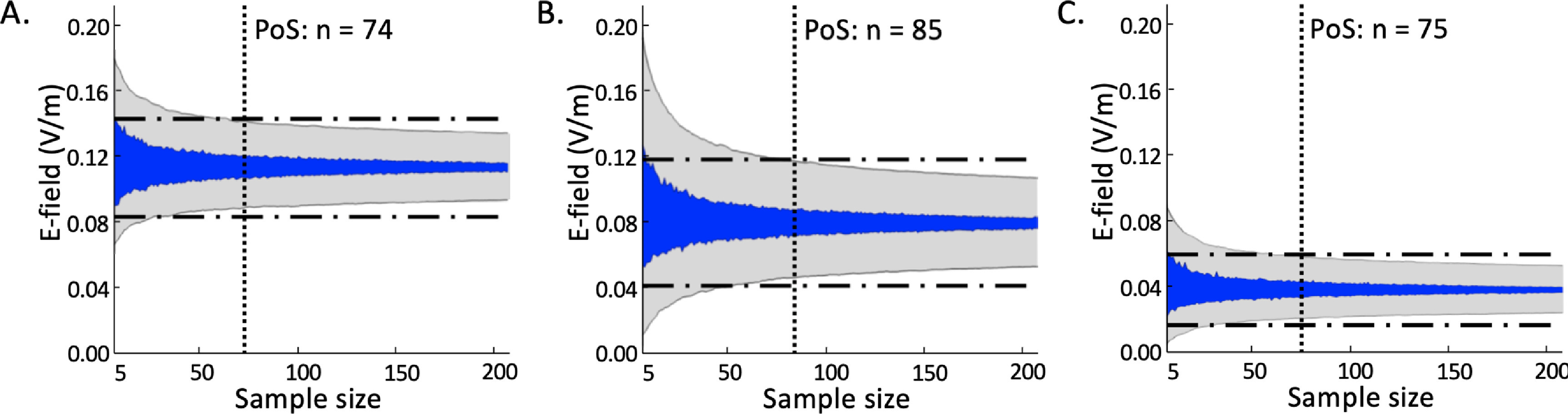
Bootstrapped group average electric field (E-field) strengths (blue area) for (A) conventional, (B) 4 × 1, and (C) center-surround transcranial electrical stimulation. Horizontal dashed lines show the corridor of stability, defined as the 5th and 95th percentile range of the total sample (n = 289). The point of stability (PoS) is the point where the group average E-field ± standard deviation (grey area) enters the corridor of stability and does not leave it at increasing subsample sizes.

In line with figure 3, conventional M1-SO tES required a sample size of 74 participants to achieve a stable group-average E-field strength. For the 4 × 1 tES montage, the point of stability was achieved at 85 participants. Using center-surround tES, the point of stability was achieved at 75 participants. As such, S1 consisted of 85 participants. The group average ROI E-field strength induced in S1 was 0.110 V/m for conventional tES, 0.079 V/m for 4 × 1 tES and 0.035 V/m for center-surround tES. These group averages were used for intensity individualization in S2.

### 3.2. Fixed versus individual transcranial electrical stimulation intensity in an independent subsample 2

**Figure 4.**
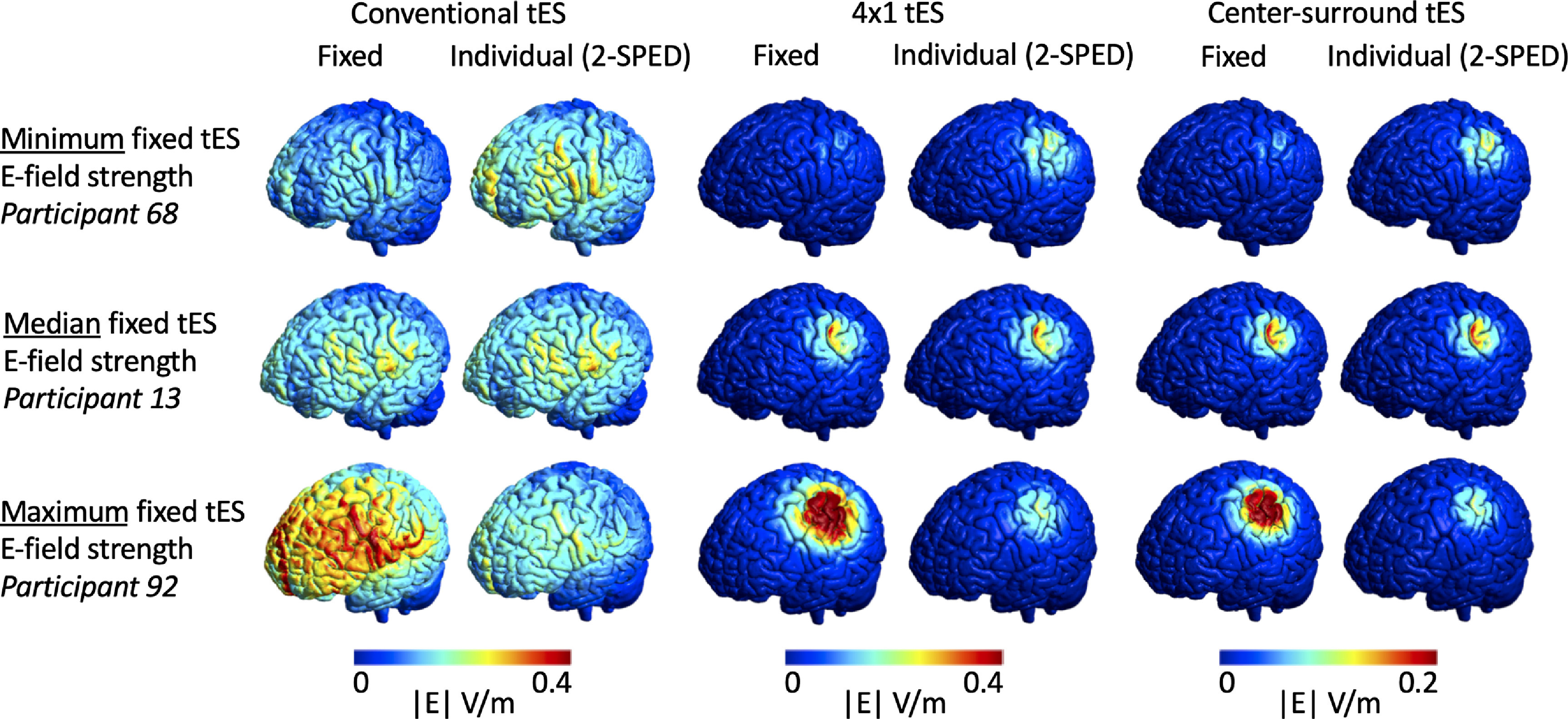
Qualitative comparison of electric fields (E-fields) induced by conventional-, 4 × 1- and center-surround transcranial electrical stimulation (tES). Each row shows one participant, namely the participant with the minimum (row 1), median (row 2) and maximum (row 3) E-field strength induced by fixed intensity tES across all 3 montages. Fixed intensity tES induces highly variable E-field strengths across participants while individual intensity tES [i.e. the 2-sample prospective dosing (2-SPED) approach] induces nearly identical E-field strengths across participants.

In S2, individual stimulation intensity to match the group average ROI E-field strengths from S1 ranged between 0.549–1.498 mA (Conventional M1-SO tES), 0.309–2.307 mA (4 × 1 tES) and 0.331–2.190 mA (Center-surround tES) (tables 1 and 2). In figure 4, we compared the E-fields produced by the individual stimulation intensity to the fixed stimulation intensity at 1 mA. Visually, fixed intensity tES induces highly variable E-field strengths across participants, while individual intensity tES effectively eliminates these variations. Although not within the scope of the current work, figure 4 also suggests that the focality of the induced E-fields became more similar across participants.

**Table 1.** Electric fields strength induced in the region of interest (the primary motor cortex, M1).

Montage	tES intensity (mA)	Average ± SD (V/m)	Variance coefficient (%)	Minimum (V/m)	Maximum (V/m)
Conventional M1-SO tES	Fixed (1)	0.112 ± 0.020	17.54	0.072	0.200
	Individual (0.549–1.498)	0.110 ± 0.00	0.00	0.110	0.110
4 × 1 tES	Fixed (1)	0.080 ± 0.027	33.96	0.034	0.255
	Individual (0.309–2.307)	0.079 ± 0.00	0.00	0.079	0.079
Center-surround tES	Fixed (1)	0.038 ± 0.013	33.54	0.016	0.105
	Individual (0.331–2.190)	0.035 ± 0.00	0.00	0.035	0.035

SD = standard deviation, SO = contralateral supraorbital area, tES = transcranial electrical stimulation.

**Table 2.** Peak cortical electric fields strength.

Montage	tES intensity (mA)	Average ± SD (V/m)	Variance coefficient (%)	Minimum (V/m)	Maximum (V/m)
Conventional M1-SO tES	Fixed (1)	0.208 ± 0.029	14.16	0.145	0.338
	Individual (0.549–1.498)	0.206 ± 0.019	9.36	0.164	0.251
4 × 1 tES	Fixed (1)	0.094 ± 0.031	32.85	0.038	0.250
	Individual (0.309–2.307)	0.092 ± 0.007	7.46	0.077	0.110
Center-surround tES	Fixed (1)	0.043 ± 0.014	32.49	0.016	0.091
	Individual (0.331–2.190)	0.040 ± 0.003	8.61	0.030	0.049

M1 = primary motor cortex, SD = standard deviation, SO = contralateral supraorbital area, tES = transcranial electrical stimulation.

### 3.3. Conventional transcranial electrical stimulation

**Figure 5.**
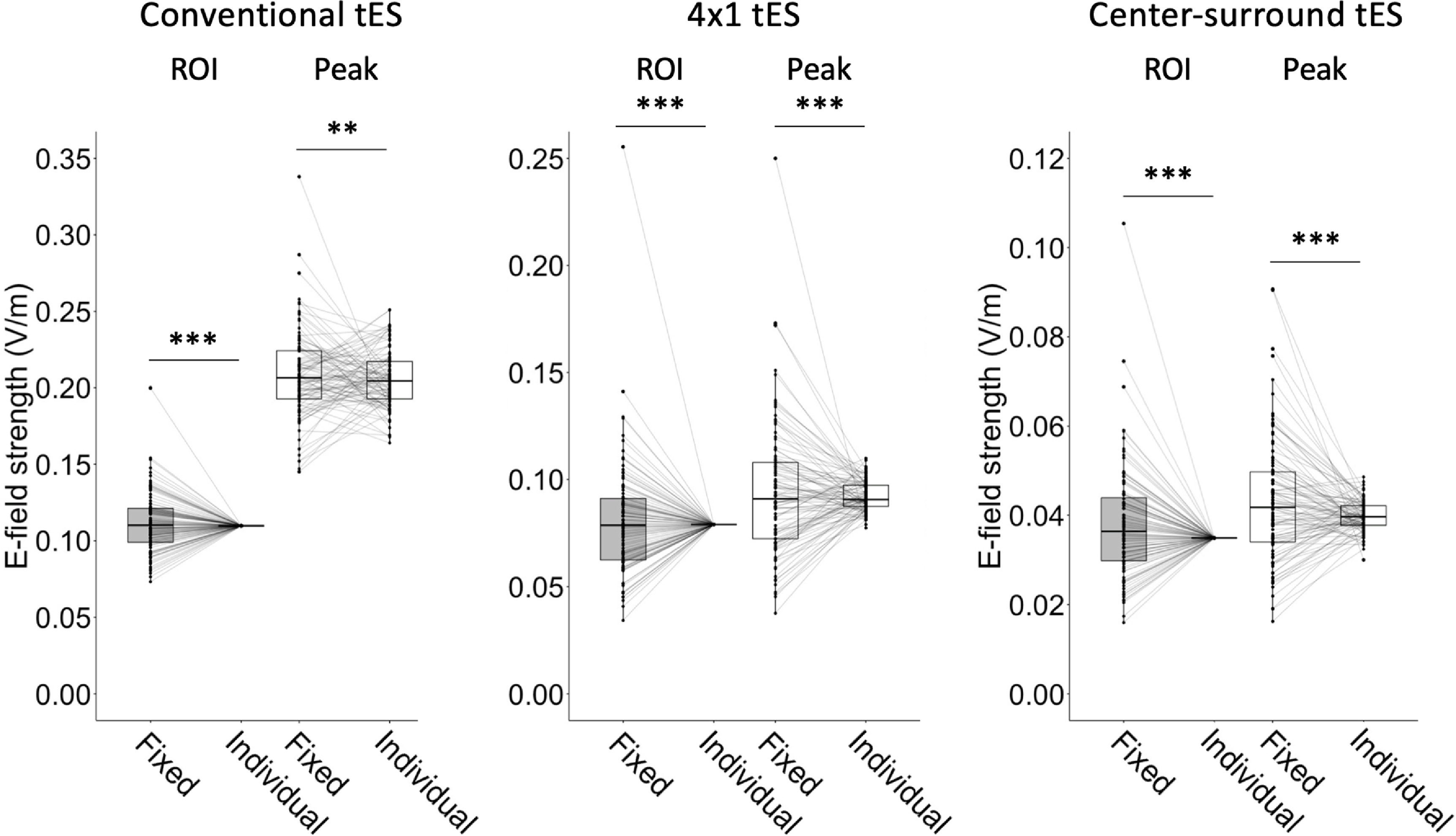
Average E-field strength in the region of interest (ROI) (grey boxplots) and peak cortical E-field strength (white boxplots) induced by fixed and individual intensity transcranial electrical stimulation (tES). Conventional- (left graph), 4 × 1- (middle) and center-surround (right) tES are shown. Variability across participants decreased significantly when using individual intensity tES. **, *** indicate a significant difference in variability as tested by the modified Pitman–Morgan test, with p < 0.01 and p < 0.001, respectively.

While fixed tES induced an E-field strength of 0.112 ± 0.020 V/m in the ROI, individualized tES induced an E-field strength of 0.110 ± 0 V/m. Variability in ROI E-field strength induced by fixed tES (VC = 17.54%, range = 0.128 V/m) was significantly higher than variability induced by individualized tES (VC = 0%, range = 0 V/m), *r*_98_ = 1, p < 0.001 (figure 5).

Peak E-field strength induced by fixed and individual tES was 0.208 ± 0.029 V/m and 0.206 ± 0.019 V/m, respectively. Variation in peak E-field strength induced by fixed tES (VC = 14.16%, range = 0.193 V/m) was significantly higher than variation induced by individualized tES (VC = 9.36%, range = 0.087 V/m), *r*_98_ = 0.26, p = 0.009 (figure 5).

### 3.4. 4 × 1 transcranial electrical stimulation

Fixed tES induced an E-field strength of 0.080 ± 0.027 V/m in M1, while individualized tES induced an E-field strength of 0.079 ± 0 V/m. Variation in M1 E-field strength induced by fixed tES (VC = 33.96%, range = 0.221 V/m) was significantly higher than variation induced by individualized tES (VC = 0%, range = 0 V/m ), *r*_98_ = 1, p < 0.001 (figure 5).

Peak E-field strength induced by fixed and individualized tES were 0.094 ± 0.031 V/m and 0.092 ± 0.007 V/m, respectively. Variation was significantly higher as a result of fixed tES (VC = 32.85%, range = 0.212 V/m) versus individualized tES (VC = 7.46%, range = 0.033 V/m), *r*_98_ = 0.86, <0.001 (figure 5).

### 3.5. Center-surround transcranial electrical stimulation

Fixed tES-induced E-field strength was 0.038 ± 0.013 V/m in the ROI, while individualized tES induced an average E-field strength of 0.035 ± 0 V/m. Variance in M1 E-field strengths induced by fixed tES (VC = 33.54%, range = 0.089 V/m) was significantly higher than variance induced by individualized tES (VC = 0%, range = 0 V/m), *r*_98_ = 1, p < 0.001 (figure 5).

Peak E-field strength induced by fixed and individualized tES was 0.043 ± 0.014 V/m and 0.040 ± 0.003 V/m, respectively. Variation was significantly higher as a result of fixed tES (VC = 32.49%, range = 0.075 V/m) versus individualized tES (VC = 8.61%, range = 0.019 V/m), *r*_98_ = 0.87, <0.001 (figure 5).

## 4. Discussion

Here, we set out to establish the 2-SPED approach to reduce interindividual E-field strength variability. By doing so, we aimed to ameliorate the capacity of tES to instigate consistent neurophysiological and behavioral changes in the fields of basic and applied sciences. Specifically, we simulated three common tES montages in 289 healthy persons. In line with previous intracranial and computational studies, we found that E-field strengths induced by 1 mA tES remain well-below 0.5 V/m and are highly variable [13, 19, 43–45]. To illustrate the latter point, the highest ROI E-field strength induced by conventional M1–SO fixed intensity tES in a participant (0.200 V/m) was 177.78% higher than the lowest induced E-field strength (0.072 V/m). Furthermore, E-field strengths induced by fixed intensity 4 × 1 tES were the most variable (cf, figure 2 and table 2), which corrobarates previous work stating that the enhanced focality of 4 × 1 HD-tES comes at the cost of increased inter-individual variability [46]. The 2-SPED approach significantly reduced both ROI and peak E-field variabilty in all 3 tES montages. Moreover, individual stimulation intensity ranged between 0.309 and 2.307 mA across all individuals of sample 2 to produce the group average that 1 mA stimulation produces in sample 1. As such, it remained well-within the proposed tES intensity safety limits [47, 48].

While the current simulations were restricted to 1 mA tES, the implications of our findings are not. Our results are extrapolatable to other stimulation intensities such as 2 and 4 mA tES due to the linear ohmic nature of tES E-field generation and the linearity of the 2-SPED method [21, 44]. For instance, by multiplying all E-field strength (V/m) values by 2, one acquires the values that 2 mA tES simulations would obtain. This would not affect the statistical results given that all values would be multiplied by the same factor.

Several methods have been developed to diminish interindividual tES E-field strength variability via stimulation intensity individualization, with none of the approaches being empiricaly tested via *in-vivo*, physical, studies. For instance, Evans *et al* introduced Dose-Controlled tES, which reverse-calculates an individual stimulation intensity [19]. Another approach uses the transcrianal electrical stimulation (TES) induced motor threshold for individualization [21]. Finally, Antonenko *et al* individualized stimulation intensity to obtain more uniform E-field strengths across individuals through head circumference measurements [49]. Although all of these methods yield merit, they are subject to several limitations which limit their implementability. For instance, practical implementation of Dose-Controlled tES is hindered by its need to scan an entire study sample, prior to being able to individualize tES. By facilitating prospective use (i.e. one could scan the first participant and do individualized tES on the same day), 2-SPED solves this. Also, TES induced motor threshold individualization is limited by its inability to measure E-field strength and by the fact that TES motor threshold determination can be intolerable for some individuals. Finally, the head circumference individualization approach only explains ∼25% of the variance of E-field strengths. Therefore, the approach still permits a substantial degree of interindividual E-field strength variability. As 2-SPED addresses these limitations, implementation is more feasible. This focus on feasibility compliments the necessity of the scientific field to start empirically researching the effect of individualized intensity tES on neurophysiological and behavioral parameters.

Conceptualization and validation of our novel 2-SPED tES approach facilitates the use of the reverse-calculation method in a prospective, *in-vivo* manner. Results indicate that it is possible to calculate a stable group average E-field value from a dataset (S1), and use the obtained value for individualisation of tES intensity in a second sample (S2), that is yet to be recruited. As such, our 2-SPED approach allows researchers to determine and implement an informed group average E-field strength for prospective dosing using reverse-calculation E-field modeling. However, it is important to note that the group average E-field strength of S1 is not necessarily the optimal E-field strength to induce maximal physiological and/or behavioral effects and is limited by the tES intensities applied to date (typically 2 mA or below). Several studies have associated higher E-field strengths with greater neurophysiological and behavioral improvements, either directly or indirectly (through higher stimulation intensities which give rise to higher E-field strengths) [10–13, 15, 50, 51]. At first glance, this seems to imply that inducing high E-field strengths is more advantageous then inducing group average E-field strengths. Although this might hold true, one should be cautious portraying the relationship between tES induced E-field strength and neurophysiological/behavioral effects as unilinear. First, this assumption does not consider factors such as stimulation duration, despite the fact that stimulation duration may alter the longevity of the induced effects and even influences the direction of tES-instigated effects [52, 53]. Second, this hypothesis contradicts the results of Batsikadze *et al* and Weller *et al*, who demonstrated that higher tES stimulation intensities (i.e. with higher induced E-fields) can shift the direction of neural effects and can reduce the effectiveness of tES in terms of cognitive improvements [22, 54]. Third, as most tES studies have delivered stimulation at intensities of 2 mA or lower, the dose-response curve has not yet been fully elucidated. It could be that a certain point, the potential benefit of increasing stimulation intensity (∼E-field strength) reaches a plateau. From this point onwards, further increasing stimulation intensity will only result in elevated participant discomfort and should therefore be avoided. Thus, the potential benefit of using 2-SPED and basing individualized E-field dosing on group average E-fields is that it theoretically ensures that participants are neither under- nor over-stimulated, as is the case in conventional fixed intensity tES.

To advance the field of noninvasive brain stimulation, it is of vital importance that future studies set out to unravel the optimal E-field strength through dosage titration. While previous studies have aimed to achieve this through comparisons of different stimulation intensities and/or post-hoc correlations linking induced E-field strength to the outcome measure, we propose that that the 2-SPED approach could be equally valuable. Implementation of 2-SPED would ensure that all participants receive nearly-identical E-fields in the targeted region. Thus, the risk of underdosing certain participants would be minimized. In parallel, by reducing E-field variability inherent to fixed-intensity tES, 2-SPED allows researchers to better isolate the impact of different tES parameters that also determine tES effectiveness, without conflating these changes with different inter-individual E-fields. Lastly, in contrast to post-hoc correlational studies linking E-field strength to outcome measures, the 2-SPED approach is capable of delivering causal evidence for the presence of an optimal E-field strength. In doing so, it can also confirm the importance of E-field strength as a vital parameter of tES, and the relevance of computational E-field dosimetry.

A potential avenue for a future study aiming to achieve these goals could be to first determine a group average E-field strength in an available participant cohort (or use a group average reported here, if participant and tES characteristics are corresponding) and prospectively use reverse-calculations to induce 0.5×, 1×, and 2× the group average E-field in a second population, comparing neurophysiological, behavioral and/or clinical effects of the different E-fields. If an optimal E-field strength were to be identified, the reverse-calculation method could be used to induce this E-field strength in all participants, irrespective of anatomy.

## 5. Limitations

The current work was subject to several limitations that should be considered.

First and foremost, while there is strong evidence in favor of the link between E-field strength and tES outcome [8–15], factors such as tES duration, tES timing (i.e. online versus offline administration), brain state and applied current direction also determine tES effectiveness [18, 55–57]. Given that these factors are not individualized via 2-SPED, one can expect 2-SPED to not entirely mitigate tES outcome variability. However, given that 2-SPED controls for the important variable E-field strength, it provides a more controlled approach to disentangle these other factors in the future. For instance, investigating how tES duration influences tES effectiveness becomes much more straightforward when one is certain that all participants receive the same E-field strength at the neural ROI. Moreover, as there is reason to believe that E-field strength and tES duration are non-linearly related, the appeal of inducing uniform E-field strengths to investigate tES duration in a more controlled manner increases even further [58, 59].

Second, 2-SPED has not yet been validated *in-vivo*. Although the advantages of 2-SPED in computational models and its feasibility are promising, empirical evidence via neurophysiological and behavioral experiments, is the much-needed next step prior to implementing 2-SPED in routine practice. This could be a promising avenue for future studies.

Third, our approach assumes that E-field simulations are accurate. While most intracranial recordings support this assumption [43, 44, 60, 61], misestimations have been observed [60]. Thus, when applying the 2-SPED method in an experimental setting, one should be aware that there might be some individuals who do not receive the same E-field strength as the rest of the group due to misestimations. Nevertheless, 2-SPED is compatible with updates to E-field methodology. Future improvements in E-field simulation accuracy will lead to a decreased number of misestimations and will further ameliorate the use of 2-SPED. Furthermore, despite some misestimations, the 2-SPED approach should still significantly improve E-field homogeneity across persons, on average. On a similar note, simulations are dependent on MRI-scan parameters. Therefore, future work should aim to acquire scans in line with the current best-practice, unless a strong rationale is present to deviate from them [27–29]. Likewise, the accuracy of the simulations, and thus by extension 2-SPED, depends on the accuracy of tissue conductivity values. Here, we used standard conductivity values, which have been used by a previous tES modeling validation study [60]. Nevertheless, it is important to acknowledge that tissue conductivity uncertainty impacts the accuracy of E-field simulations [62]. Incorrect tissue conductivity values in some individuals could lead to misestimations by the 2-SPED approach, which, in turn, could cause 2-SPED to fail its goal of inducing uniform E-fields in these individuals.

Fourth and finally, we opted to use SimNIBS—headreco (SPM12 + CAT12) for modeling and segmentation, although several other approaches are available (i.e. CHARM, ROAST and SimNIBS—mri2mesh) [33, 34, 63]. While an elaborate comparison of these approaches is beyond the scope of our work and has already been conducted [33, 63], it is important to emphasize that post-segmentation processing in SimNIBS—headreco enforces all tissue layers to be fully enclosed by the subsequent tissue layer, prioritizing continuity of layers at the cost of anatomical accuracy. On the other hand, the SimNIBS—headreco approach enables the inclusion of accurate surface segmentations of the brain pial surfaces into the model building process, which is not possible via approaches that rely only on the anatomically coarser results of volume segmentation methods. Moreover, SimNIBS 3 creates one homogenous bone tissue layer with a single, adjusted, conductivity value. Although the anatomically correct, three-layered bone tissue model (i.e. spongious bone enclosed by compact bone on the interior and exterior side of the skull) has been incorporated in some head models, a single bone layer with adjusted conductivity value yields similar E-field strength-related results, is less computationally demanding, and is considerably more often used in the E-field modeling literature [29, 43, 44, 60–62, 64, 65].

## 6. Conclusion

Through three commonly used tES montages, we demonstrated that the 2-SPED approach enables prospective individualization of tES intensity to induce uniform E-field strengths in a population. In line with previous literature, we demonstrated that individual intensity tES produces identical E-fields in the ROI across participants, as well as significantly less variable peak cortical E-fields. Given that E-field strength is a cardinal physical agent of tES, 2-SPED yields great scientific promise. It can be implemented to unravel the neural effects underlying tES, and to investigate other determinants of tES effectiveness, such as current direction and stimulation duration, while controlling for a large source of variability (i.e. E-field strength).

## Data Availability

The data that support the findings of this study are available from the corresponding author upon reasonable request.
